# Validation of computer self-administered screening and assessment tools to identify unhealthy substance use in medical patients

**DOI:** 10.1186/1940-0640-10-S1-A38

**Published:** 2015-02-20

**Authors:** Jennifer McNeely, Shiela Strauss, Charles M Cleland, John Rotrosen, Arianne Ramautar, Derek Nelsen, Marc N Gourevitch

**Affiliations:** 1 Department of Population Health, NYU School of Medicine, New York, NY, 10016, USA; 2 NYU College of Nursing, New York, NY, 10003, USA; 3 Department of Psychiatry, NYU School of Medicine, New York, NY, 10016, USA

## Background

Substance use frequently goes undetected in medical settings, in part due to lack of an efficient approach to screening and assessment. To address this, we developed computer self-administered tools, which could be completed prior to the medical encounter, to screen for unhealthy use of tobacco, alcohol, illicit drugs, and prescription drugs.

## Methods

We developed a four-item screener called the Substance Use Brief Screen (SUBS), and a brief assessment that is an audio computer-assisted self-interview version of the Alcohol, Smoking, and Substance Involvement Screening Test (ACASI ASSIST). Adult patients were recruited consecutively from a safety net primary care clinic. Participants completed the SUBS and ACASI ASSIST in English using a touchscreen tablet computer, followed by interviewer-administered reference standard measures. We evaluated the sensitivity and specificity of the SUBS for detecting past-year unhealthy substance use, and the concordance of ACASI ASSIST responses with the previously validated interviewer-administered ASSIST.

## Results

Among the 390 participants, prevalence of unhealthy substance use was 31 percent for tobacco, 17 percent for alcohol, and 22 percent for other drugs. Sensitivity and specificity of the SUBS for detecting past year unhealthy use were: tobacco 99 percent and 91 percent (AUC = .95); alcohol 94 percent and 68 percent (AUC = .81); and drugs (illicit or prescription) 86 percent and 89 percent (AUC = .87). Sensitivity was lower for prescription drugs (56%) than for illicit drugs (80%). The ACASI ASSIST demonstrated excellent concordance (92–99%) with the ASSIST in identifying moderate to high-risk substance use, though illicit drug use was more frequently reported on the ACASI ASSIST. The median time required to complete the ACASI ASSIST was 3.7 minutes (range 0.7–15.4), and 53 (13.5%) participants required assistance using one or both tools. Eighty-five percent of participants said they either preferred the computer to an interviewer, or had no preference.

## Conclusions

The SUBS and ACASI ASSIST appear to offer an accurate, feasible, and acceptable approach to identifying unhealthy substance use in primary care patients. The SUBS has sufficient sensitivity and specificity to identify patients who have unhealthy alcohol or drug use, and thus require further assessment. Assessment can be reliably performed by the ACASI ASSIST. Both instruments could be completed by patients either in the waiting area or from home using a portal into the electronic health record, with results delivered to the primary care provider at the point of care. Applied in this way, the SUBS and ACASI ASSIST have the potential to ease barriers to implementing substance use screening and assessment in medical settings.

